# Nonlinear associations between sleep duration and the risks of all-cause and cardiovascular mortality among the general adult population: a long-term cohort study

**DOI:** 10.3389/fcvm.2023.1109225

**Published:** 2023-06-14

**Authors:** Jie Li, Qiyong Wu, Li Fan, Zining Yan, Dan Shen, Ming Zhang

**Affiliations:** ^1^ Department of Echocardiography, ChangZhou No.2 People’s Hospital Affiliated to Nanjing Medical University, Changzhou, China; ^2^ Cardio-Thoracic Surgery, ChangZhou No.2 People's Hospital Affiliated to Nanjing Medical University, Changzhou, China

**Keywords:** NHANES, sleep duration, CVD, mortality, all-cause

## Abstract

**Objective:**

This study aims to investigate the correlation between sleep duration and all-cause and cardiovascular mortality in the general population.

**Methods:**

A total of 26,977 participants aged ≥18 years were included in the analysis from the National Health and Nutrition Examination Survey (NHANES) database covering the period from 2005 to 2014. Data on cardiovascular and all-cause deaths were collected until December 2019. Sleep duration was assessed using a structured questionnaire, and participants were categorized into five groups based on their reported sleep duration (≤5, 6, 7, 8, or ≥9 h). Kaplan-Meier survival curves were employed to examine the mortality rates across different sleep duration groups. Multivariate Cox regression models were utilized to explore the association between sleep duration and mortality. Additionally, a restricted cubic spline regression model was employed to identify the non-linear relationship between sleep duration and all-cause and cardiovascular mortality.

**Results:**

The average age of participants was 46.23 ± 18.48 years, with 49.9% of the subjects being male. Over a median follow-up period of 9.42 years, 3,153 (11.7%) participants died from all-cause mortality, among which 819 (3.0%) were attributed to cardiovascular causes. The groups with sleep durations of ≥9 and ≤5 h exhibited the lowest cumulative survival rates for all-cause mortality and cardiovascular mortality, respectively. When using a sleep duration of 7 h as the reference, the hazard ratios (with 95% confidence intervals) for all-cause mortality were 1.28 (1.14–1.44) for ≤5 h, 1.10 (0.98–1.23) for 6 h, 1.21 (1.10–1.34) for 8 h, and 1.53 (1.35–1.73) for ≥9 h. The hazard ratios (with 95% confidence intervals) for cardiovascular mortality were 1.32 (1.04–1.67) for ≤5 h, 1.22 (0.97–1.53) for 6 h, 1.29 (1.05–1.59) for 8 h, and 1.74 (1.37–2.21) for ≥9 h. A U-shaped non-linear relationship between sleep duration and all-cause and cardiovascular mortality was observed, with inflection point thresholds at 7.32 and 7.04 h, respectively.

**Conclusion:**

The findings suggest that the risk of all-cause and cardiovascular mortality is minimized when sleep duration is approximately 7 h.

## 1. Introduction

Sleep is a fundamental physiological process that plays a critical role in maintaining human health and overall well-being. It serves as a natural adaptation to the alternation of day and night, regulated by intricate neural mechanisms and following a strict rhythm (1). However, the fast-paced nature of modern life has resulted in significant changes in sleep patterns, posing a significant public health concern. Recent research has placed considerable emphasis on sleep duration and its impact on cardiovascular health (1).

Previous studies have highlighted associations between sleep duration and the prevalence of cardiovascular disease (CVD), suggesting a potential link between insufficient sleep and an elevated risk of CVD and all-cause mortality (2). Some researchers have even proposed sleep duration as a potential predictor of cardiovascular outcomes (3). Additionally, inadequate sleep duration has been linked to a higher risk of developing or dying from coronary heart disease (CHD) and stroke, although not for total CVD (4). On the other hand, an increased risk of CHD, stroke, and total CVD has been associated with long sleep duration (4). Both short and long sleep durations serve as predictors or markers and are associated with a heightened risk of all-cause mortality and cardiovascular events (4, 5). Nevertheless, studies focusing on healthy individuals (6) and middle-aged participants (7) have reported no significant association or correlation between sleep duration and mortality. It is crucial to consider various factors such as differences in research objectives, study designs, sample sizes, follow-up periods, and confounding variables that may influence these findings. Consequently, further investigations are warranted to comprehensively evaluate the relationship between sleep duration and all-cause and cardiovascular mortality.

Given the existing gaps in knowledge, our study aims to explore the association between sleep duration and all-cause and cardiovascular mortality using data obtained from a large cohort of participants included in the National Health and Nutrition Examination Survey (NHANES). As an observational study, our research contributes to the expanding body of evidence on this topic and provides valuable insights into the potential relationship between sleep duration and cardiovascular outcomes.

## 2. Materials and methods

### 2.1. Study population and design

The National Center for Health Statistics, a division of the Centers for Disease Control and Prevention, has conducted a series of cross-sectional surveys in the United States known as NHANES since the 1960s. NHANES adheres to the guidelines outlined in the Helsinki Declaration, and all research-related agreements for this survey have undergone review and approval. Written informed consent was obtained from all participants. The NHANES database, containing the information utilized in this study, is publicly accessible online at no cost.

**Figure 1:**
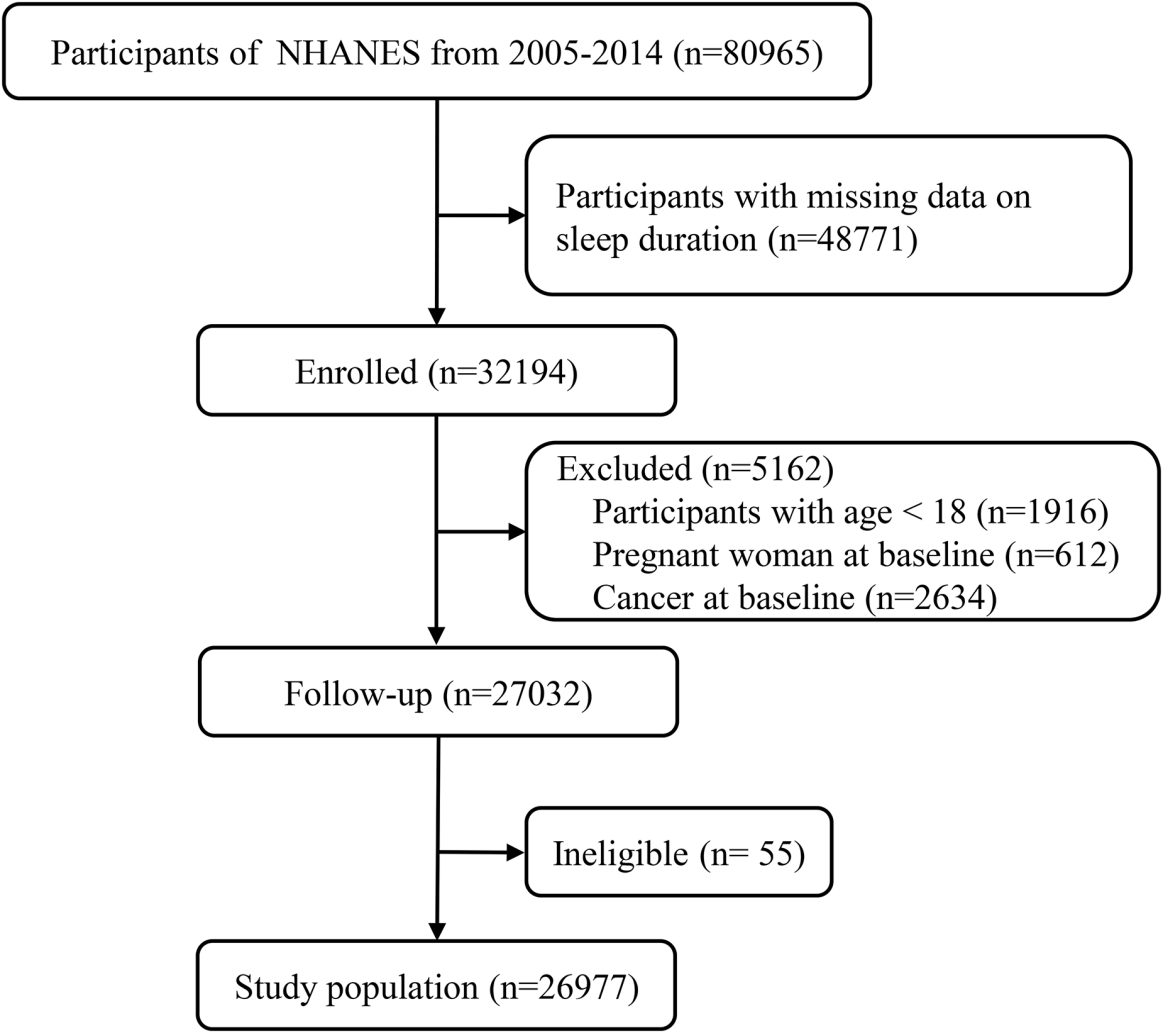
Flow chart of the study.

For our study, we analyzed data from a total of 80,965 respondents who participated in surveys conducted between 2005 and 2014. We excluded 48,771 participants due to the absence of sleep duration data, 1,916 participants under the age of 18 years, 612 participants with baseline pregnancy, and 2,634 participants with baseline cancer. The remaining 27,032 participants were tracked for survival until December 2019. During the follow-up period, 55 respondents were excluded due to missing or incomplete data. Ultimately, our final dataset included data from 26,977 respondents (Figure 1).

### 2.2. Assessment of sleep duration

Sleep pattern data were collected during NHANES surveys conducted from 2005 to 2014. Each participant completed a structured questionnaire that inquired about their sleep and waking times on weekdays and rest days, as well as any nocturnal awakenings. Sleep duration was determined using the following question: “How much sleep do you usually get at night on weekdays or workdays?” Participants provided the number of hours of sleep they typically obtained on a typical night, which was recorded as a continuous variable. Based on recommendations from the American Academy of Sleep Medicine, sleep duration was categorized as follows: ≤5, 6, 7, 8, or ≥9 h (8). The definition was based on the “NHANES Questionnaire” data on “sleep disorders,” and further comprehensive information can be found at the following website: https://wwwn.cdc.gov/Nchs/Nhanes/2013-2014/SLQ_H.htm#SLD010H.

### 2.3. Mortality outcome assessment

Mortality data for this study were extracted from publicly available NHANES mortality documents from 2005 to 2014 and subsequently matched with records from the National Death Index (NDI) to verify mortality status. The primary endpoints assessed in this study were all-cause mortality and cardiovascular mortality. All-cause mortality encompassed death from any cause, while cardiovascular mortality referred to deaths caused by cardiovascular or cerebrovascular disease, according to the International Classification of Diseases, Tenth Revision (ICD-10) (9).

### 2.4. Data collection

Data from 2005 to 2014 were collected in a standardized manner from structured documents, data sets, and questionnaires included in the NHANES database. Demographic information including age, sex, race (Mexican American, Other Hispanic, Non-Hispanic White, Non-Hispanic Black, or Other Race), and education level (no high school diploma, high school diploma, or education beyond high school diploma) was collected for all participants. Body mass index (BMI) values were obtained (10), and participants were classified into the following categories: underweight (BMI <18.5), healthy weight (BMI 18.5–24.9), overweight (BMI 25–29.9), or obese (BMI ≥30). Hypertension was defined as a systolic blood pressure ≥140 mmHg or a diastolic blood pressure ≥90 mmHg. Participants taking antihypertensive medications or with a known history of hypertension were also considered hypertensive (11). Data regarding total cholesterol, fasting blood glucose, and glycosylated hemoglobin levels were collected, and the glomerular filtration rate (GFR) was estimated. The presence of diabetes was defined as a fasting blood glucose level ≥126 mg/dl or a hemoglobin A1c value ≥6.5%. Participants who self-reported having diabetes or used hypoglycemic medications were also considered diabetic (12). The GFR was calculated using the standard kidney disease formula (13). Participants with CVD were defined as those who had been diagnosed with at least one of the following conditions: CHD, congestive heart failure, angina pectoris, myocardial infarction, or stroke. Other covariates assessed included physical activity, smoking status, and drinking status. Physical activity levels were classified as inactive (no physical activity), insufficiently active (some physical activity), or active (≥5 sessions of moderate exercise or ≥3 sessions of strenuous exercise per week) (14). Smoking status was categorized as never smokers (participants who have smoked <100 cigarettes during their lifetime), former smokers (participants who have smoked >100 cigarettes but have quit smoking), or current smokers (participants who have smoked >100 cigarettes and currently smoke) (15). Drinking status was classified as nondrinker, low-to-moderate drinker (<2 drinks/day in men and <1 drink/day in women), or heavy drinker (≥2 drinks/day in men and ≥1 drinks/day in women) (16).

### 2.5. Statistical analysis

Data analysis and graphical visualization were performed using R project (version 4.1.1). Continuous variables with a normal distribution were reported as mean ± standard deviation (SD), while continuous variables with a non-normal distribution were presented as median (quartile interval). Categorical variables were expressed as numbers and percentages. Kaplan-Meier (KM) curves were generated to illustrate the relationship between sleep duration and all-cause and cardiovascular mortality.

To assess the impact of sleep duration on mortality, multivariate Cox proportional hazard regression analysis was conducted. Hazard ratios (HR) and 95% confidence intervals (CI) were calculated for different sleep durations. The assumption of proportional hazards required by the Cox model was evaluated using Schoenfeld residuals, and no violations were observed. Additionally, variance inflation factors (VIF) were calculated to examine multicollinearity among the independent variables in the multiple Cox model. Three multivariate Cox regression models were constructed with different adjustments of covariates. Model 1 included adjustments for age, sex, and race. Model 2 included the covariates from Model 1 and further adjusted for education level, drinking status, smoking status, BMI, and physical activity. Model 3 incorporated the covariates from Models 1 and 2, with additional adjustments for total cholesterol level, GFR, and the presence of hypertension, diabetes, and CVD. A sleep duration of 7 h served as the reference category. To explore the potential nonlinear association between sleep duration and all-cause mortality as well as cardiovascular mortality, a multivariate adjusted Cox restricted cubic spline regression model was employed. If a nonlinear relationship was observed, the threshold point was estimated and further explored. Statistical significance was defined as *p*-values <0.05.

## 3. Results

### 3.1. Population characteristics

A total of 26,977 participants (49.9% male) were included in the final dataset, with a mean age of 46.23 ± 18.48 years. Among the participants, 41.3% were classified as Non-Hispanic White, 50.1% had an education level beyond a high school diploma, 71.0% were categorized as low to moderate drinkers, and 40.5% were considered physically active. Out of all participants, 8,545 (31.7%) were never smokers. The prevalence of hypertension, diabetes, and CVD was 31.7%, 10.8%, and 9.1%, respectively. The mean total cholesterol level was 192.17 ± 41.74 mg/dl, and the mean estimated GFR was 96.26 ± 24.28 ml/min/1.73 m^2^ (Table 1).

**Table 1:** Baseline characteristics of adults with hypertension in NHANES 1999–2014.

Characteristics	Total	Sleep duration, h/day				
		≤5	6	7	8	≥9
Participants, *n*	26,977	4,149	6,354	7,161	7,164	2,149
Age, years	46.23 (18.48)	47.20 (17.35)	45.70 (17.36)	45.21 (17.87)	46.51 (19.31)	48.38 (22.26)
Male, %	13,472 (49.9)	2,093 (50.4)	3,232 (50.9)	3,650 (51.0)	3,519 (49.1)	978 (45.5)
Race/ethnicity, %						
Mexican American	4,601 (17.1)	541 (13.0)	1,041 (16.4)	1,241 (17.3)	1,404 (19.6)	374 (17.4)
Other Hispanic	2,515 (9.3)	430 (10.4)	586 (9.2)	640 (8.9)	677 (9.5)	182 (8.5)
Non-Hispanic White	11,148 (41.3)	1,401 (33.8)	2,428 (38.2)	3,281 (45.8)	3,055 (42.6)	983 (45.7)
Non-Hispanic Black	6,124 (22.7)	1,431 (34.5)	1,660 (26.1)	1,207 (16.9)	1,385 (19.3)	441 (20.5)
Other race	2,589 (9.6)	346 (8.3)	639 (10.1)	792 (11.1)	643 (9.0)	169 (7.9)
Education level, %						
Below high school	7,309 (27.1)	1,245 (30.0)	1,550 (24.4)	1,668 (23.3)	2,127 (29.7)	719 (33.5)
High school	6,161 (22.8)	1,052 (25.4)	1,482 (23.3)	1,523 (21.3)	1,611 (22.5)	493 (22.9)
Above high school	13,507 (50.1)	1,852 (44.6)	3,322 (52.3)	3,970 (55.4)	3,426 (47.8)	937 (43.6)
Drinking status, %						
Nondrinker	5,850 (21.7)	942 (22.7)	1,245 (19.6)	1,445 (20.2)	1,667 (23.3)	551 (25.6)
Low-to-moderate drinker	19,163 (71.0)	2,905 (70.0)	4,684 (73.7)	5,194 (72.5)	4,960 (69.2)	1,420 (66.1)
Heavy drinker	1,964 (7.3)	302 (7.3)	425 (6.7)	522 (7.3)	537 (7.5)	178 (8.3)
Smoking status, %						
Never	15,282 (56.6)	2,050 (49.4)	3,583 (56.4)	4,274 (59.7)	4,189 (58.5)	1,186 (55.2)
Former	5,812 (21.5)	887 (21.4)	1,294 (20.4)	1,574 (22.0)	1,592 (22.2)	465 (21.6)
Current	5,883 (21.8)	1,212 (29.2)	1,477 (23.2)	1,313 (18.3)	1,383 (19.3)	498 (23.2)
BMI status, %						
Underweight	510 (1.9)	62 (1.5)	114 (1.8)	111 (1.6)	158 (2.2)	65 (3.0)
Normal	7,959 (29.5)	1,031 (24.8)	1,747 (27.5)	2,260 (31.6)	2,240 (31.3)	681 (31.7)
Overweight	8,748 (32.4)	1,287 (31.0)	2,095 (33.0)	2,368 (33.1)	2,365 (33.0)	633 (29.5)
Obese	9,760 (36.2)	1,769 (42.6)	2,398 (37.7)	2,422 (33.8)	2,401 (33.5)	770 (35.8)
Physical activity, %						
Inactive	6,945 (25.7)	1,136 (27.4)	1,526 (24.0)	1,564 (21.8)	1,929 (26.9)	790 (36.8)
Insufficiently active	9,100 (33.7)	1,263 (30.4)	2,142 (33.7)	2,618 (36.6)	2,392 (33.4)	685 (31.9)
Active	10,932 (40.5)	1,750 (42.2)	2,686 (42.3)	2,979 (41.6)	2,843 (39.7)	674 (31.4)
Total cholesterol, mg/dl	192.17 (41.74)	193.58 (43.89)	191.61 (41.14)	192.60 (41.10)	192.55 (41.85)	188.45 (40.84)
eGFR, ml/min/1.73 m^2^	96.25 (24.28)	96.26 (24.24)	97.23 (23.12)	96.49 (22.96)	96.23 (24.95)	92.56 (29.00)
Hypertension, %	8,545 (31.7)	1,641 (39.6)	2,067 (32.5)	1,938 (27.1)	2,150 (30.0)	749 (34.9)
Diabetes, %	2,909 (10.8)	580 (14.0)	667 (10.5)	601 (8.4)	749 (10.5)	312 (14.5)
CVD, %	2,463 (9.1)	517 (12.5)	520 (8.2)	474 (6.6)	649 (9.1)	303 (14.1)
Follow-up time, years	9.37 (3.17)	9.36 (3.18)	9.46 (3.09)	9.57 (3.08)	9.29 (3.18)	8.80 (3.51)

Normally distributed continuous variables are described as means ± SEs. Categorical variables are presented as numbers (percentages). BMI, body mass index; eGFR, estimated glomerular filtration rate; CVD, cardiovascular disease.

### 3.2. Relationships between sleep duration and mortality

During a median follow-up of 9.42 years, a total of 3,153 (11.7%) participants died from all-cause mortality, including 819 (3.0%) cardiovascular deaths. The three multivariate Cox regression models consistently showed the association between sleep duration and all-cause and cardiovascular mortality across various subgroups (Table 2).

**Table 2:** Hrs (95% CIs) of all-cause and cardiovascular mortality according to sleep duration among adults in NHANES 2005–2014.

Range	Sleep duration, h/day				
	≤5	6	7	8	≥9
All-cause mortality					
No. deaths/total	544/4,149	614/6,354	614/7,161	919/7,164	462/2,149
Model 1	1.49 (1.32–1.67)	1.15 (1.03–1.29)	1.00 [Reference]	1.29 (1.17–1.43	1.86 (1.65–2.10)
Model 2	1.35 (1.20–1.51)	1.11 (0.99–1.25)	1.00 [Reference]	1.24 (1.12–1.37	1.64 (1.45–1.85)
Model 3	1.28 (1.14–1.44)	1.10 (0.98–1.23)	1.00 [Reference]	1.21 (1.10–1.34	1.53 (1.35–1.73)
Cardiovascular mortality					
No. deaths/total	137/4,149	161/6,354	144/7,161	241/7,164	136/2,149
Model 1	1.57 (1.24–1.99)	1.28 (1.02–1.60)	1.00 [Reference]	1.39 (1.13–1.71)	2.14 (1.69–2.71)
Model 2	1.40 (1.11–1.78)	1.24 (0.99–1.56)	1.00 [Reference]	1.32 (1.08–1.63)	1.87 (1.48–2.38)
Model 3	1.32 (1.04–1.67)	1.22 (0.97–1.53)	1.00 [Reference]	1.29 (1.05–1.59)	1.74 (1.37–2.21)

Model 1 was adjusted as age, sex, and race; Model 2 was adjusted as model 1 plus education level, drinking status, smoking status, BMI, and physical activity; Model 3 was adjusted as model 2 plus total cholesterol, eGFR, hypertension, diabetes, and cardiovascular disease.

**Figure 2:**
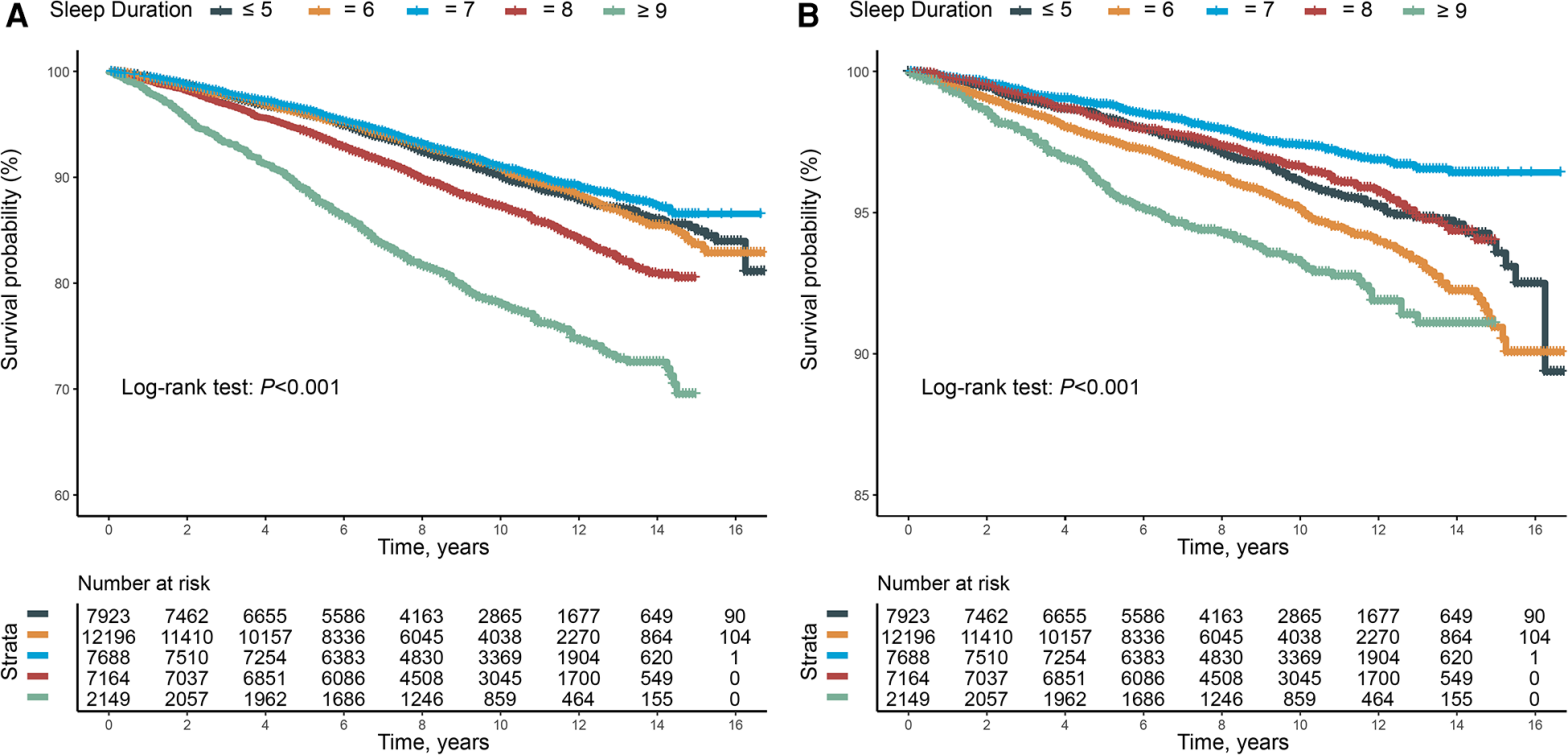
(**A**) Kaplan–Meier survival curve for all-cause mortality. (**B**) Kaplan–Meier survival curve for cardiovascular mortality.

Overall, the group with sleep duration ≥9 h exhibited the highest risk for all-cause mortality (log-rank *P* for trend vs. reference <0.001) (Figure 2A). Across all three models, both longer sleep durations (≥9 and 8 h) and shorter sleep durations (≤5 h) were associated with a higher risk of all-cause mortality (Table 2).

Similarly, sleep durations longer or shorter than the reference of 7 h were associated with an increased risk of cardiovascular mortality (log-rank *P* for trend vs. reference <0.001) (Figure 2B). Consistent with the findings for all-cause mortality, both longer sleep durations (≥9 and 8 h) and shorter sleep durations (≤5 h) were associated with an increased risk of cardiovascular mortality across all three models (Table 2).

### 3.3. Nonlinear relationships between sleep duration and mortality

**Figure 3:**
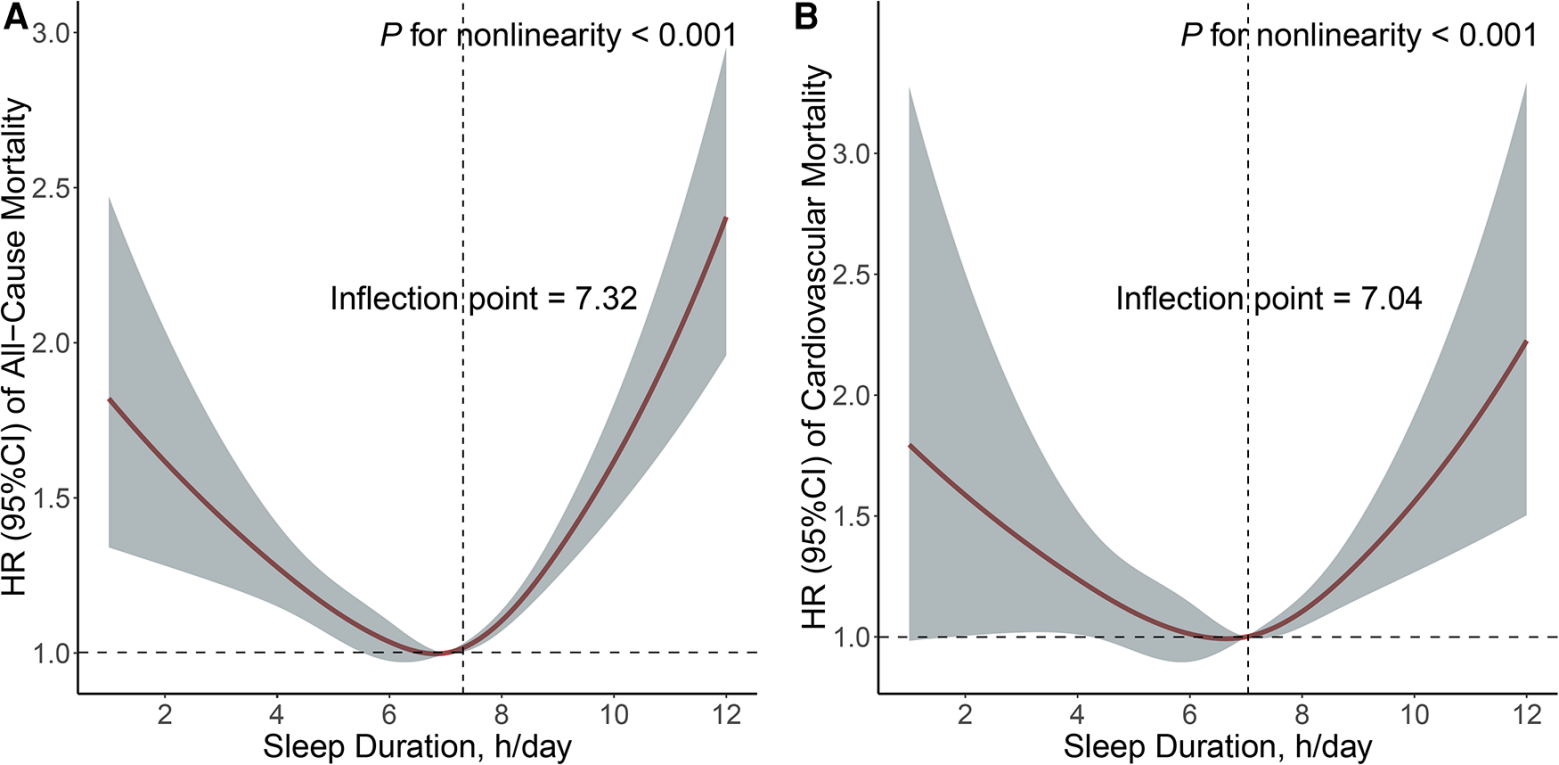
Non-linear relationship between sleep duration and all-cause (**A**) and cardiovascular (**B**) mortality. Adjusted for age, sex, and race, education level, drinking status, smoking status, BMI, physical activity, total cholesterol, GFR, hypertension, diabetes, and CVD.

A U-shaped association was observed between sleep duration and both all-cause mortality (Figure 3A) and cardiovascular mortality (Figure 3B). The hazard ratio (HR) for all-cause mortality decreased to the lowest point when sleep duration was 7.32 h and increased significantly with longer sleep duration. The threshold for sleep duration in relation to cardiovascular mortality was 7.04 h. The HR for cardiovascular mortality showed a downward trend when sleep duration was less than 7.04 h and an upward trend when sleep duration exceeded 7.04 h, although the upward trend was not as pronounced as that observed for all-cause mortality.

## 4. Discussion

In this large, long-term study, we observed that the optimal sleep duration associated with the lowest risk of all-cause and cardiovascular mortality was approximately 7 h. These results confirm the findings from a previous cross-sectional study of sleep duration and cardiovascular health (17) and reinforce the detrimental impact of sleep disorders on cardiovascular health.

The American Academy of Sleep Medicine, the Sleep Research Society, and the National Sleep Foundation define “short sleep” as a duration of less than 7 h per night (18, 19). Although there is some variation in the definitions used across studies, it is important to note that our results align with the recommendation of 7 h of sleep per night as outlined in a 2015 consensus statement by the American Academy of Sleep Medicine and the Sleep Research Society (20). This statement emphasized that sleep durations below 7 h can impair immune function, affect metabolism, and have adverse effects on cardiovascular, cerebrovascular, and mental health. Furthermore, insufficient sleep has been associated with increased risks of obesity, diabetes, hypertension, heart disease, stroke, depression, and mortality (20). Several population-based cohort studies have consistently shown that individuals with shorter sleep durations are at higher risk of all-cause and cardiovascular death (21–23). Our findings reinforce these previous observations, indicating that a sleep duration of approximately 7 h represents a threshold for increased risk, with significantly shorter sleep durations corresponding to higher mortality risk.

Researchers have made efforts to elucidate the underlying mechanisms linking short sleep duration and increased cardiovascular risk. One potential mechanism involves metabolic dysregulation associated with inadequate sleep. Abnormalities in the release of peptides and leptin, which are involved in regulating energy intake, may contribute to this process. Peptides stimulate hunger and increase appetite, while leptin inhibits hunger and reduces appetite (24). Recent studies have demonstrated that shorter sleep durations can promote peptide release and inhibit leptin release, leading to increased food intake, weight gain, decreased insulin sensitivity, and elevated blood glucose levels, ultimately contributing to obesity and impaired glucose tolerance (25). Sleep duration has also been correlated with lipid metabolism. A recent study revealed a significant nonlinear association between short sleep duration and low high-density lipoprotein (HDL) levels and high triglyceride levels (26). Another proposed mechanism involves the neuro regulation of central body tissues. In individuals with insufficient sleep, increased stimulation of the cardiovascular sympathetic nerves results in heightened tension, reduced parasympathetic activity, elevated heart rate, and heightened reactivity, ultimately leading to increased blood pressure and vascular endothelial cell damage (27). Additionally, a study found that short sleep duration leads to decreased blood flow velocity in the cerebral basilar artery and midbrain, resulting in regional inadequate blood supply to the brain and triggering oxidative stress, inflammation, platelet dysfunction, and metabolic disorders (28).

The definition of “long sleep” is not well-defined, similar to “short sleep.” The consensus statement from the American Academy of Sleep Medicine and the Sleep Research Society stated that it remains uncertain whether sleep durations exceeding 9 h per night are associated with health risks for the general population, excluding individuals who are ill or have missed sleep (7). Recent Mendelian randomization studies have suggested a potential causal link between short sleep duration and CVD, but not for long sleep duration (29, 30). While our study found an increased risk of all-cause and cardiovascular mortality among individuals who slept more than 7 h per night, even after adjusting for covariates, we acknowledge that our findings do not establish a causal relationship. It is important to consider confounding factors in observational studies, such as conditions like depression, antidepressant use, or poor sleep quality, which can lead to longer sleep duration. These factors may confound the observed association, and the increased risk of CVD associated with long sleep duration could reflect underlying health conditions or residual confounding rather than a direct causal relationship.

The potential mechanisms underlying the association between long sleep duration and increased cardiovascular risk have also been investigated. An analysis of data from the Korea National Health and Nutrition Examination Survey (KNHANES) demonstrated a significant correlation between sleep duration of 9 h or more and low high-density lipoprotein cholesterol (HDL-C) levels (31). Another analysis found a positive correlation between longer sleep durations and increased levels of C-reactive protein (CRP) and interleukin-6 (IL-6) (32). In women, CRP levels were approximately 44% higher in those with habitual sleep duration of 9 h or more compared to those with 8 h of sleep after adjusting for age and BMI (33). CRP production is stimulated by IL-6, suggesting that the correlation between sleep duration and CRP levels may be secondary to the effect of sleep duration on IL-6 levels. Increased levels of CRP and IL-6 have previously been associated with an elevated risk of CVD and diabetes. Therefore, similar to short sleep duration, one potential mechanism by which long sleep duration may impact cardiovascular health is through the activation of inflammatory pathways. Insulin resistance may also play a role in the association between long sleep duration and cardiovascular risk. A study of nurses found that regular sleep durations exceeding 9 h were associated with a 47% increased risk of diabetes and a 57% increased risk of cardiac events (34, 35). Another recent study reported an increased risk of all-cause mortality among adult patients with type 2 diabetes who slept more than 7 h per night (36). Insulin resistance is a key risk factor for diabetes, and long sleep duration may contribute to an increased risk of insulin resistance through factors such as lack of physical activity, chronic inflammation, and disturbance of circadian rhythm. Furthermore, a prospective analysis of elderly individuals in China revealed that excessive sleep duration was associated with an increased risk of cognitive impairment, suggesting the need for early attention to neurodegeneration (37). Lastly, prolonged sleep duration may be associated with factors such as undiagnosed chronic conditions, depression, unemployment, low socioeconomic status, and reduced physical activity, which could further contribute to increased cardiovascular morbidity and mortality in these individuals (38).

A recent study found that both short sleep and long sleep were associated with an increased 10-year risk of CHD (39). However, a previous meta-analysis demonstrated that long sleep was more strongly correlated with adverse outcomes than short sleep, and the association between long sleep and mortality was not attenuated by improvements in health or lifestyle (40). In our study, the steep increase in the curve for all-cause mortality compared to cardiovascular mortality may be attributed to undiagnosed chronic complications among participants. This finding is highly consistent with previous studies that have reported an association between long sleep duration and increased mortality, particularly for non-cardiovascular cause (41, 42). Some researchers have even proposed using long sleep duration as a diagnostic tool for detecting subclinical or undiagnosed physical and mental disorders (43).

Our study had several strengths, including a relatively large sample size and a long follow-up period. However, it is important to acknowledge certain limitations inherent in our research. Firstly, while we were able to quantitatively measure the duration of sleep, the overall assessment of sleep is multifaceted and involves various aspects such as circadian rhythm, sleep initiation and maintenance difficulties, and sleep-related breathing disorders. Moreover, it is important to note that sleep patterns may change over time. Our study relied on a single assessment of sleep patterns at baseline, and we did not collect follow-up data to capture potential changes in sleep duration or quality during the study period. These aspects were not comprehensively captured in our study, which may have limited our understanding of the complexity of sleep patterns and their potential effects on health outcomes. Secondly, our study relied on self-reported sleep data, which introduces the possibility of memory biases and inaccuracies in reporting. Objective measures of sleep, such as polysomnography or actigraphy, could provide more precise and detailed information on sleep patterns and help overcome these limitations. Finally, while we made efforts to control for numerous confounding factors, it is possible that unmeasured confounders may exist. It is worth highlighting that our study did not include laboratory investigations such as inflammatory markers or hormone levels. As a result, our observations regarding potential underlying mechanisms remain largely speculative, and further research incorporating these measures is warranted to provide a more comprehensive understanding of the relationships between sleep duration and health outcomes.

## 5. Conclusion

In this large-scale, long-term study, we have further clarified the exact relationship between sleep duration and all-cause and cardiovascular mortality in American adults, demonstrating that the risk of all-cause and cardiovascular mortality was lowest when sleep duration was approximately 7 h. However, the pathophysiological mechanisms linking sleep disorders and negative prognosis require further investigation. Future studies should focus on understanding the underlying mechanisms and determining whether interventions targeting both sleep duration and quality can effectively reduce morbidity and mortality rates. These studies will be crucial in developing strategies to improve sleep-related health outcomes.

## Data Availability

The original contributions presented in the study are included in the article, further inquiries can be directed to the corresponding author.
